# Pol II promoter prediction using characteristic 4-mer motifs: a machine learning approach

**DOI:** 10.1186/1471-2105-9-414

**Published:** 2008-10-04

**Authors:** Firoz Anwar, Syed Murtuza Baker, Taskeed Jabid, Md Mehedi Hasan, Mohammad Shoyaib, Haseena Khan, Ray Walshe

**Affiliations:** 1 Department of Computer Science and Engineering, East West University, Bangladesh; 2 Department of Genetic Engineering and Biotechnology, University of Dhaka, Bangladesh; 3 Institute of Information Technology, University of Dhaka, Bangladesh; 4 Department of Biochemistry and Molecular Biology, University of Dhaka, Bangladesh; 5 Biocomputation Research Group, Dublin City University, Ireland

## Abstract

**Background:**

Eukaryotic promoter prediction using computational analysis techniques is one of the most difficult jobs in computational genomics that is essential for constructing and understanding genetic regulatory networks. The increased availability of sequence data for various eukaryotic organisms in recent years has necessitated for better tools and techniques for the prediction and analysis of promoters in eukaryotic sequences. Many promoter prediction methods and tools have been developed to date but they have yet to provide acceptable predictive performance. One obvious criteria to improve on current methods is to devise a better system for selecting appropriate features of promoters that distinguish them from non-promoters. Secondly improved performance can be achieved by enhancing the predictive ability of the machine learning algorithms used.

**Results:**

In this paper, a novel approach is presented in which 128 4-mer motifs in conjunction with a non-linear machine-learning algorithm utilising a Support Vector Machine (SVM) are used to distinguish between promoter and non-promoter DNA sequences. By applying this approach to plant, Drosophila, human, mouse and rat sequences, the classification model has showed 7-fold cross-validation percentage accuracies of 83.81%, 94.82%, 91.25%, 90.77% and 82.35% respectively. The high sensitivity and specificity value of 0.86 and 0.90 for plant; 0.96 and 0.92 for Drosophila; 0.88 and 0.92 for human; 0.78 and 0.84 for mouse and 0.82 and 0.80 for rat demonstrate that this technique is less prone to false positive results and exhibits better performance than many other tools. Moreover, this model successfully identifies location of promoter using TATA weight matrix.

**Conclusion:**

The high sensitivity and specificity indicate that 4-mer frequencies in conjunction with supervised machine-learning methods can be beneficial in the identification of RNA pol II promoters comparative to other methods. This approach can be extended to identify promoters in sequences for other eukaryotic genomes.

## 1 Background

A promoter is a signal element on a DNA molecule that specifies a controlling region of a gene where RNA polymerase binds to initiate the transcription of the gene. RNA polymerase II (RNA pol II) in eukaryotic cell binds the promoter signals of all protein-coding sequences. As there is no common collective occurrence of these signals it is extremely difficult to predict the promoter efficiently using an arbitrary or small set of predetermined signals. The approach adopted in this research considers a much larger set of signals that are not mutually exclusive and increases the success rate of finding a promoter in a given sequence significantly. A number of methods for the prediction of promoters, TSS (Transcription Start Signals) and TF (Transcription Factor) binding sites in eukaryotic DNA sequence presently exist [1,2].

Although contemporary algorithms have improved on predictive ability, a lot more research is required to achieve satisfactory levels of performance. Many general-purpose promoter prediction implementations can typically recognize only ~50% of the promoters with a false positive (FP) rate of ~1 per 700–1000 bp [2]. A stepwise strategy method CorePromoter based on localization of functional promoter and TSS region was suggested by Zhang. [3] The use of Markov chain models in promoter prediction tools by Ohler et al. [4] improved the results slightly but they identified the same 50% of promoters from the dataset analyzed by Fickett and Hatzigeorgiou [2], while having a false positive prediction rate of 1/849 bp. Later Ohler [5] improved the result by incorporating some prominent motifs such as TATA, DRE, INR, DPE, MTE [6] to discriminate promoter and non-promoter using Hidden Markov Model. Also, another promoter identification program, Promoter 2.0, designed by Knudsen [7] applied a combination of neural networks and genetic algorithms. Promoter 2.0 was experienced on recognizing promoters in the complete Adenovirus genome (35,937 bp). The program predicted some known promoter sites such as TATA box, cap site, CCAAT box and GC box on the positive strand and 30 false promoters. Pandey *et. al*. [8] analyzed plant promoters in terms of their structural and sequence dependent properties like curvature and periodicity. Promoters with TATA box and without TATA box have also been analyzed in this regard.

Since completion of the sequencing of the human genome, the efficiency of promoter prediction tools became critical and still poses a major challenge. PromoterInspector program [9] was the first software tool used to identify the promoters in human chromosome 22. This method identifies ~50% of known promoters as genomic regions up to 1 kb in length by discriminating them from the exon, intron and 3'-untranslated region (3'-UTR) sequences. Davuluri et al. proposed FirstEF program which included a decision tree based on structural and compositional discriminating features such as CpG islands, promoter regioins and first splice-donor sites [10]. In 2002, Bajic et al. [11] reported the Dragon Promoter Finder (DBF) program [12], which used sensors for three functional regions: promoters, exons and introns. Their findings claimed that DBF has higher accuracy than three other promoter finding programs which they had investigated namely: NNPP 2.1 [13-15] Promoter2.0 [7,16]and PromoterInspector [17]. Also in 2002, Down and Hubbard [18] reported a novel hybrid machine-learning method capable of predicting > 50% of human TSS with a specificity of > 70%. Xiao et. al. [19] predicted Pol II promoter sequence by cooperating Transcription Factor Binding Site (TFB) with the promoter sequence. They have identified that nearly 71% of the promoter sequences contain transcription factor with known cooperation. Rajeev et. al. [20] proposed another promoter identification tool where they identified the promoter sequence from non promoter sequence using non linear time series descriptor using non linear machine learning algorithm, such as SVM. Their approach showed 87% accuracy in 10 fold cross-validation test with an independent test having accuracy of near 85% in identifying promoter and non-promoter. Yet another promoter prediction tool, TSSP-TCM has been trained and adapted for plants [21]. In the test set of TATA promoters, the program correctly predicted TSS for 35 out of 40 (87.5%) genes. For 25 TATA-less promoters, TSSs were predicted for 21 out of 25 (84%) genes, including 14 cases of 5 bp distance between annotated and predicted TSSs. A different approach was undertook by Gershenzon and Ioshikhes, besides identifying core promoter elements in Human they tried to demonstrate the synergistic effect of the combination of several core promoter elements for the initiation of the transcription [22]. While Jin et al. later in their study revealed a three-way synergistic effect using a degree of conservation between orthologous mouse and human sequence [23].

The proposed method in this paper demonstrates that with the aid of PromMachine- a machine-learning tool, promoters are distinguished from the non-promoter sequences on the basis of abundance of some characteristic 4-mer motifs. The PromMachine is trained with 128 distinguishing 4-mers that can discriminate between promoter and non promoter. Using this knowledge the machine-learning tool can efficiently decide whether a given sequence contains a promoter or not.

With high sensitivity, specificity and accuracy the proposed approach shows high efficiency in promoter prediction in eukaryotic genomic sequences. Being applicable for any reasonable length (251 bp length of a promoter is considered to be a reasonable length) of given sequences this approach can become a dynamic tool for finding promoters of other eukaryotes.

## 2 Results and discussion

### 2.1 Prediction Accuracy

Different statistical measures have been used to analyze the performance of the proposed model. In order to test the prediction accuracy, 50 known promoter sequences from each species i.e. human, mouse, plant, rat and Drosophila as well as 50 known non-promoter sequences for each of those five species are initially taken for the test. Then taking one species at a time i.e. 50 known promoter and 50 known non-promoter for that species, the previously trained model is applied and tested upon these test data. It is to be mentioned that these test dataset are completely independent from the training set. In most of the case the model correctly predicts the promoters and non-promoter in these test data. Sensitivity and specificity are two widely used techniques for performance evaluation, which are defined as following, where TP is true positive, FN is false negative, FP is false positive and TN is truly negative.

(1) *Sensitivity *= *TP*/(*TP *+ *FN*)

(2) *Specificity *= *TN*/(*FP *+ *TN*)

The results of sensitivity and specificity for all the species are recorded in Table 1. As it can be seen from Table 1 a sensitivity of 86% and specificity of 90% for plant; 96% and 92% for Drosophila; 88% and 92% for human; 78% and 84% for mouse and finally 82% and 80% for rat are achieved, which demonstrates the high performance of the proposed model for correctly identifying a promoter as a promoter and a non-promoter as a non-promoter. Also it demonstrates that, the model is less prone to false positive and false negative. Besides sensitivity and specificity, a number of other statistical procedures such as correlation coefficient (Corr.), false positive rate (FPR), false negative rate (FNR), positive predictive value (PPV), negative predictive value (NPV), positive likelihood (PL) and negative likelihood (NL) are also calculated for each of the species. The FPR values for each species show that the proportions of negative instances that are erroneously reported as being positive are low.

**Table 1:** Predictive accuracy calculation of the proposed model using different statistical measures

**Predicted Sequences**	**TP**	**FP**	**FN**	**TN**	**Sn**	**Sp**	**Corr.**	**FPR**	**FNR**	**PPV**	**NPV**	**PL**	**NL**
Plant Promoter	43	Nil	7	Nil	0.86	0.90	0.761	0.10	0.14	0.89	0.86	8.60	0.15
									
Plant CDS/Non-Promoter	Nil	5	Nil	45									

Drosophila Promoter	48	Nil	2	Nil	0.96	0.92	0.881	0.08	0.04	0.92	0.95	12	0.04
									
Drosophila CDS/Non-Promoter	Nil	4	Nil	46									

Human Promoter	44	Nil	6	Nil	0.88	0.92	0.801	0.08	0.12	0.92	0.88	11	0.13
									
Human CDS/Non-Promoter	Nil	4	Nil	46									

Mouse Promoter	39	Nil	11	Nil	0.78	0.84	0.621	0.16	0.22	0.83	0.79	4.87	0.26
									
Mouse CDS/Non-Promoter	Nil	8	Nil	42									

Rat Promoter	41	Nil	9	Nil	0.82	0.80	0.620	0.20	0.18	0.80	0.81	4.10	0.22
									
Rat CDS/Non-Promoter	Nil	10	Nil	40									

The PPV values in Table 1 shows that about 80% times a promoter is correctly identified as promoter in rat, whereas this accuracy increased to 83% in mouse, 89% in plant and 92% for both Drosophila and human. However, because the PPV depends on the prevalence of the dataset, a same proportion of positive and negative data are taken for the test. The likelihood ratios are also calculated, as it is independent of the prevalence (Table 1).

Cross validation is another statistical process for estimating the predictive accuracy of a classifier on data. In this process, the whole dataset i.e. both positive and negative data for a given species is partitioned into subsets in such a way that the analysis is made on one subset while all the other subsets are used for training. To analyze the performance of some other n-mers; first, 3, 4 and 5-mers are generated and tested against the same dataset. A cross-validation accuracy of 81.78%, 83.81% and 83.27% are obtained, which justifies the use of 4-mer motif for proposed promoter prediction methodology. A differential hexamer technique was used earlier by Hutchinson for identifying vertebrate promoter; on 29 test sequences he correctly distinguished 18 promoters as true positive whereas 11 were false positive, which gave him a Sensitivity of 62.1%. The result improved up to 71.4% when only sequences of length above 10,000 were considered [24]. Also Chan and Kibler have used 6-mer distribution for identifying cis-regulatory motifs in Drosophila and obtained a Sensitivity and Specificity of 38.68%, 93.77%, and the PPV was 36.05% [25], which is significantly improved upon by the method proposed in this paper (Table 1). This supports the significance of 4-mer distribution used here compared to other n-mers. The rational of 4-mer giving better results is, for lower or higher mers the frequency distribution of the feature set are not descriptive enough for the SVM classifier to distinguish promoters from the non-promoters. Moreover a significant overrepresentation of 4-mer motif such as ATAA, TATA or TCAG was confirmed earlier by Ohler et al. [26], it may also speculate the justification of 4-mer performing better than some other n-mers.

**Figure 1:**
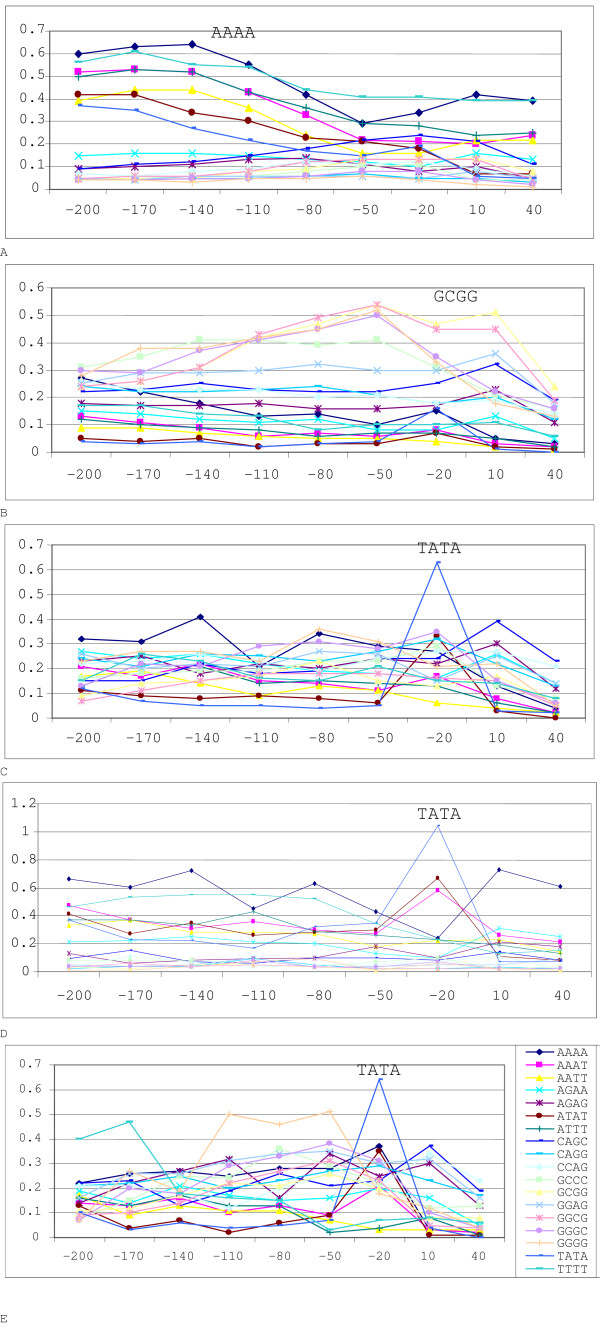
Top 18 motifs distribution along with position in Drosophila (A), Human (B), Mouse (C), Plant (D) and Rat (E).

When the model presented in the paper is trained with the 128 discriminating 4-mer features of a given species and tested; the results from the model are promising. A 7-fold cross validation for all five species displays accuracy of 83.81%, 94.82%, 91.25%, 90.77% and 82.35% for plant, Drosophila, human, mouse and rat respectively (Table 2). The high-percentage of correct value, correlation coefficient for this proposed model clearly indicates that calculated frequencies of 4-mer sequences are capable of discriminating with high accuracy between promoter and non-promoter regions. The top five 4-mers which are most frequent in the promoter dataset of all five species are listed in Table 3. A union of these motifs across the species results in a total of 18 motifs all together. The positional distributions of these 18 motifs for each of the five species are shown in Figure 1. Although measuring the conservation of these 4-mer motifs across different species is outside the scope of this research but positional conserveness of the 18 motifs in each five species i.e. where in the sequence dataset for a given species these 18 motifs mostly exists, can be observed from the figures presented. Also the top 10 discriminating features (motifs) between plant promoter and plant non-promoter are listed in Table 4.

**Table 2:** Cross validation accuracy

**Algorithm Applied to**	**7-Fold Cross Validation Accuracy**
Plant	83.81%
Drosophila	94.82%
Human	91.25%
Mouse	90.77%
Rat	82.35%

**Table 3:** Top five 4-mer motifs in different species arranged in order, highest on the top

**Rank**	**Drosophila**	**Human**	**Mouse**	**Plant**	**Rat**
1	TTTT	GCGG	AAAA	AAAA	GGAG
2	AAAA	GGCG	GGGG	TTTT	GGGG
3	ATTT	GGGG	CAGG	AAAT	AGAG
4	AAAT	GGGC	CCAG	TATA	CAGG
5	AATT	GCCC	GGGC	ATAT	CAGC

**Table 4:** The top 10 most discriminating 4-mer sequences found within promoter and non-promoter region for plant

**Rank**	**Frequency Difference**	**4-mer**
1	564	TATA
2	438	ATAT
3	435	AAAA
4	431	ATAA
5	407	TAAA
6	373	GAAG
7	324	TTTT
8	312	ATAA
9	302	AGAA
10	302	GGAA

In addition, the proposed method identifies the location of the TATA box using TATA weighted matrix [27]. For this a sliding window technique is used with a window size of 12 and cutoff score of the matrix is selected as 7.16. Each time the window is shifted by 1 bp across the sequences. The windows with a TATA box score exceeding the cutoff score are accepted as TATA Box. This indicates the possible location of promoter. As TATA box is usually located 25 base pairs upstream to the transcription start site (TSS), using this information TSS can also be identified in the TATA Box containing promoter sequences.

### 2.2 Comparison with existing methods

Similarity based methods such as BLAST is normally the first choice to distinguish between two different set of data. But no significant distinguishable features are identified using promoter-promoter, non-promoter – non-promoter and promoter-non-promoter BLAST search. To date various algorithms and methods are used for promoter prediction. Using these algorithms, some widely used promoter prediction tools have been developed, e.g. Soft Berry, Dragon Promoter Finder, Neural Network Promoter Prediction, Promoter 2.0 Prediction Server and Promoter Scan.

However, the model proposed here demonstrates even better performance than these tools. The results shown in Table 5 clearly indicate that the prediction accuracy of the model is much higher in comparison to other tools. There are a number of key features in this model which contribute to the improved performance such as the use of larger sets (128) of discriminating features between promoters and non promoters and the utilisation of the supervised learning system- SVM. Other prominent promoter prediction tools use either a statistical approach or Neural Network such as SoftBerry (TSSP). However, SVM has out-performed both of these approaches in pattern matching and supervised learning. Also most of the afore-mentioned approaches are limited in the number of features used as the promoter signals such as TATA box or Initiator etc. In contrast, the model described in this paper, uses SVM to detect a greater set of signals than previously attempted to date to decide whether a sequence is a promoter or non promoter. The additional features developed in the course of this research show that a significant increase in promoter prediction can be achieved using this method.

**Table 5:** Comparison of accuracy against existing methods [** = Infinity]

**Program Used**	**NNP Threshold(0.8)**	**SoftBerry(TSSP)**	**ProScan Vers. 1.7**	**Dragan Promotor Finder Vers. 1.4**	**Promotor 2.0 Pred. Server**	**Prom-Machine**
SENSITIVITY %	68	88	0	12	0	86
SPECIFICITY %	76	90	100	100	78	90
Correlation Co-Eff.	0.44	0.78	**	0.25	**	0.77

## 3 Conclusion

The successful prediction of promoters with high accuracy using frequency distribution of 4-mer sequences shows that this novel method has significant merit as an approach for successful eukaryotic promoter prediction. The principal objective of this work was to develop an efficient tool that can discriminate between promoter and non-promoter in an unknown sequence with high accuracy. The high accuracy of promoter prediction in plant, human, Drosophila, mouse and rat (Table 2) using our approach has validated the use of frequency distribution of 4-mers in discrimination between promoter and non promoter regions in eukaryotic sequences. However, though the approach is very efficient in predicting the presence of promoter in a given sequence, it cannot locate the position of the TSS when TATA box is not present. This challenge should be resolved in future if other signals can be characterized for specific position in the sequence. The improvement of identification of all TSS site will be considered in future using various published databases such as MPromDB, TRED, DBTSS etc. Though the EPD is known to have a TATA bias [23,28], the result presented in this paper is not likely to vary much using other database, cause the proposed method does not rely on the identification of the TATA box for discriminating promoters from non-promoters. Incorporating comparative genomics certainly can extend the method. It would reduce the false discovery rate further, as at a moderate phylogenetic distance the functional elements are known to be more conserved for orthologous gene [23,29]. Nevertheless, it is suggested that the approach proposed here would be a extremely useful and an efficient tool to meet the demands of the molecular biologists.

## 4 Methods

### 4.1 Promoter Sequence Database

To accomplish the task of RNA polymerase II promoter prediction, the plant promoter dataset is taken from PlantProm database [27]. A total of 305 entries of plant promoter sequence are obtained from PlantProm DB. Also to make the model applicable to predict promoters in Drosophila, human, mouse and rat, a number of 1922, 1863, 190 and 119 promoter sequences respectively are collected from EPD (Eukaryotic Promoter Database) [30,31]. These data are used for training the proposed model and tested against mutually exclusive data from the same data source. Sequence segments from -200 to +51 relative to TSS are considered from the EPD for the experiment. On the other hand -200 to +51 segments are taken from PlantProm DB.

### 4.2 Non-Promoter Sequence Database

The non-promoter sequences are extracted from Unigene database that belongs to EMBL. CDSs are the best non promoter sequence because it contain no promoter sequences and so for every organism specific model an equal number of CDSs are usaed in the training process as the non promoter sequence.

### 4.3 Support Vector Machine

SVM, a supervised machine-learning technique has been used for discriminating between promoter and non-promoter sequences. SVM classifiers solve multi-class classification problems using the structural minimization principle. Given a training set in a vector space, SVMs can find the best decision hyper plane, which separates two classes. The quality of the decision hyper plane depends on the difference margin between the two hyperplanes defined by the SVM [32]. For a typical learning task *P*(*X*, *y*) = *P*(*y*|*X*)*P*(*X*), an inductive SVM learner aims to build a decision function. Here *X*_*i *_∈ *X *are objects of the training set and *y*_*i *_∈ {-1, 1} are their known classes.

fn: X → {-1,1} based on a training set *S *which is fn = fn(*S*_*train*_)

where *S*_*train *_= (*X*_1_, *y*_1_), (*X*_2_, *y*_2_),..., (*X*_*n*_, *y*_*n*_).

It is necessary to select a kernel function and the regularization parameter in each Binary Classifier and in this instance, the Radial Basis Function (RBF) [32] is selected as the kernel function. Because the RBF kernel nonlinearly maps samples into a higher dimensional space, it can handle cases where the relations are non linear. The SVM classification problem can be formulated in terms of a convex quadratic optimization problem as

(3) $$Max\left[\sum_{i=1}^{N}\alpha_{i}-\frac{1}{2}-\sum_{i,j=1}^{N}\alpha_{i}\alpha_{j}y_{i}y_{j}K(x_{i},x_{j})\right]$$

In the above equation *N *is the total number of input vectors, α_*i *_is any real value that maximizes the function, *x*_*i *_is any real number as the input vectors and *y*_*i *_be their corresponding target class, which is either -1 or 1 in binary classifier. LIBSVM – a library for SVM developed by Chih-Chung Chang and Chih-Jen Lin  is used to train in a supervised manner on a collection of promoter and non-promoter training sequences. The model developed from this training set is then used to predict the promoter sequences from a test sequence.

### 4.4 Procedure

#### 4.4.1 Selection of features

Taking all four nucleotides (A, T, C and G) a 4^4 ^= 256 different 4-mers combinations are generated. In order to select most discriminating features the frequency of each of these features are calculated. In this procedure the *f*_*ij *_and *fn*_*ij *_are calculated, where *f*_*ij *_is the frequency of each *i*th 4-mer motif (*i *= 1...256) in *j*th known promoter sequence and *fn*_*ij *_is the frequency of each *i*th 4-mer in *j*th known non-promoter sequence. Then this *f*_*ij *_and *fn*_*ij *_are used for calculating *P*_*i *_and *NP*_*i *_for which the equation is given below.

(4) $$P_{i}=\sum_{j=1}^{n}f_{ij}$$

(5) $$NP_{i}=\sum_{j=1}^{n}fn_{ij}$$

Where, *P*_*i *_is the summation of frequencies in n promoter sequences for each *i*th 4-mer (*i *= 1...256) and *NP*_*i *_is the summation of frequencies in n non-promoter sequences for each *i*th 4-mer. The absolute difference between the numbers of occurrences of all 256 possible combinations of 4-mers in known promoter and non-promoter sequences are calculated using *Diff*_*i *_= |*P*_*i *_- *NP*_*i *_|, where *Diff*_*i *_is the absolute difference of the occurrence of ith 4-mer between known promoter and non promoter. The value of these 256 *Diff*_*i *_are then sorted in descending order and the top 128 4-mers for which the *Diff*_*i *_is maximum are selected as features of the proposed model. All the selected n known promoter and n known non-promoter sequence are of length 251 bp. A single strand search of 251 bp window has been conducted during the frequency calculation.

#### 4.4.2 Training with SVM

The frequency *f*_*ij *_of the top 128 4-mer motifs are used to find the promoter (or non-promoter) in a given sequence. Here *i *range from 1 to 128 and *j *range from 1 to n. A scaling factor is introduced to bring the numerical value of *f*_*ij *_in between 0 and 1 using equation 4.

(6) $$d_{ij}=\frac{f_{ij}-min(f_{1},f_{2},...,f_{128})}{max(f_{1}\ldots f_{128})}$$

Here, the numerical value of *d*_*ij *_ranges from 0 to 1.

Here, the value *d*_*ij *_is the scaled value of *i*th 4-mer in *j*th sequence. A complete set of *d*_*ij *_values of all the promoter and non-promoter sequences is then used to train the SVM for a given species.

Libsvm [33], which is an efficient software for SVM classification and regression, is trained with the frequency patterns of the 128 characteristic 4-mer motifs in known promoters and non-promoters. A model is then built that can be used to distinguish between promoter and non promoter in test sequences.

#### 4.4.3 Testing

To substantiate the machine-learning model, Jackknife [34] validation could be the ideal process. Jackknife is a method that creates a series of statistics from a single data set by generating that statistic repeatedly on the data set leaving one data value out each time which produces a mean estimate of the parameter and a standard deviation of the estimates of the parameter. This validation method is time consuming. So to speed up the process, a 7-fold cross-validation is performed where the whole dataset is divided into 7 random parts and 7 iterations are performed to predict the accuracy. In every iteration 6 parts are used for training and the other part is used for testing. In the following iteration the other 6 parts are used for training and the remaining part is used for testing.

## Authors' contributions

FA was responsible for implementation and reviewing the research and manuscript. SMB was responsible for coding, implementation and manuscript preparation. TJ was responsible for implementation, coding and testing. MMH was responsible for biological information analysis and interpretation. MS proposed the idea for the research and generated the project specification. HK reviewed the research manuscript and inspired overall analysis. RW reviewed the research manuscript, revised previous versions, edited and formatted the final document.
